# Effects of Cellular Pathway Disturbances on Misfolded *Superoxide Dismutase-1* in Fibroblasts Derived from ALS Patients

**DOI:** 10.1371/journal.pone.0150133

**Published:** 2016-02-26

**Authors:** Isil Keskin, Elin Forsgren, Dale J. Lange, Markus Weber, Anna Birve, Matthis Synofzik, Jonathan D. Gilthorpe, Peter M. Andersen, Stefan L. Marklund

**Affiliations:** 1 Department of Pharmacology and Clinical Neurosciences, Umeå University, Umeå, Sweden; 2 Department of Neurology, Hospital for Special Surgery and Weill Cornell Medical Center, New York, NY, United States of America; 3 Neuromusucular Diseases Unit/ALS Clinic, Kantonsspital St.Gallen, St. Gallen, Switzerland; 4 Department of Neurodegenerative Diseases, Hertie Institute for Clinical Brain Research, Tübingen, Germany; 5 German Research Center for Neurodegenerative Diseases (DZNE), University of Tübingen, Tübingen, Germany; 6 Department of Medical Biosciences, Clinical Chemistry, Umeå University, Umeå, Sweden; Macquarie University, AUSTRALIA

## Abstract

Mutations in *superoxide dismutase-1 (SOD1)* are a common known cause of amyotrophic lateral sclerosis (ALS). The neurotoxicity of mutant *SOD1*s is most likely caused by misfolded molecular species, but disease pathogenesis is still not understood. Proposed mechanisms include impaired mitochondrial function, induction of endoplasmic reticulum stress, reduction in the activities of the proteasome and autophagy, and the formation of neurotoxic aggregates. Here we examined whether perturbations in these cellular pathways in turn influence levels of misfolded *SOD1* species, potentially amplifying neurotoxicity. For the study we used fibroblasts, which express *SOD1* at physiological levels under regulation of the native promoter. The cells were derived from ALS patients expressing 9 different *SOD1* mutants of widely variable molecular characteristics, as well as from patients carrying the GGGGCC-repeat-expansion in *C9orf72* and from non-disease controls. A specific ELISA was used to quantify soluble, misfolded *SOD1*, and aggregated *SOD1* was analysed by western blotting. Misfolded *SOD1* was detected in all lines. Levels were found to be much lower in non-disease control and the non-*SOD1* *C9orf72* ALS lines. This enabled us to validate patient fibroblasts for use in subsequent perturbation studies. Mitochondrial inhibition, endoplasmic reticulum stress or autophagy inhibition did not affect soluble misfolded *SOD1* and in most cases, detergent-resistant *SOD1* aggregates were not detected. However, proteasome inhibition led to uniformly large increases in misfolded *SOD1* levels in all cell lines and an increase in *SOD1* aggregation in some. Thus the ubiquitin-proteasome pathway is a principal determinant of misfolded *SOD1* levels in cells derived both from patients and controls and a decline in activity with aging could be one of the factors behind the mid-to late-life onset of inherited ALS.

## Introduction

Amyotrophic lateral sclerosis (ALS) is characterized by adult-onset degeneration of upper and lower motor neurons. The disease begins focally and then spreads contiguously, resulting in progressive paralysis and death from respiratory failure [1]. Mutations in the gene encoding the ubiquitously expressed free radical scavenging enzyme superoxide dismutase-1 (SOD1) are known to cause ALS [2], and are found in 1–9% of patients [3]. Since 1993, 188 coding mutations in *SOD1* have been associated with ALS as a dominant trait (http://alsod.iop.kcl.ac.uk/), but disease caused by the most prevalent *SOD1* mutation D90A is usually inherited as a recessive trait [4]. While missense mutations are most frequent, some 20 *SOD1* mutations result in insertions, deletions or substitutions resulting in C-terminal truncations or other disruptive changes, precluding native folding of the mutant protein. Importantly, there are no apparent clinical (e.g. age of onset, survival time) or post-mortem histological differences between patients carrying missense mutations and disruptive mutations [5–7]. This suggests that a common cytotoxic mechanism originates from misfolded SOD1 species. The concentrations of the most structurally stable SOD1 mutants (e.g. A89V, D90A, and L117V) are, however, similar to wild-type SOD1 in humans [8, 9]. The major proportions of these, which are natively folded and enzymatically active, are unlikely to contribute significantly to neurotoxicity. In contrast, the most disrupted truncated mutants are present at 100-fold lower levels [7, 10]. These findings suggest that minute subfractions of misfolded, not total, mutant SOD1 are the relevant pathogenic species for ALS.

The mechanisms by which misfolded SOD1 species cause the disease are poorly understood. However, they have been suggested to involve perturbation of mitochondria [11–16], induction of endoplasmic reticulum (ER)-stress [16–19], reduction of proteasome activity [20–22], reduction of autophagy [23, 24], and aggregation [25–31]. Another unresolved feature of ALS is why carriers of *SOD1* mutations are apparently healthy until late middle age, and then undergo rapid neurological decline. Typically, a carrier of a *SOD1* A4V or G93A mutation presents with a sudden focal paresis and wasting that disseminates quickly throughout the motor system, leading to death in one to two years [5, 32]. Perhaps an age-related decline in proteostasis and energy metabolism, amplified by a vicious cycle of misfolded SOD1 accumulation, leads to a rapid increase in misfolded SOD1 species in the tissue.

Studies of ALS pathogenesis involving mutant SOD1 are usually conducted in transgenic animals or transfected cell models, both of which exhibit high levels of overexpression of the mutant protein. Studies on patient material are typically conducted at end-stage. We have generated dermal fibroblast lines from ALS patients carrying mutations in *SOD1* and other ALS-linked genes and from non-disease controls. These cells, in which mutant SOD1 is expressed under the native promoter, offer opportunities for exploration which are poorly accessible in most other model systems. We have previously developed methods that enable minute amounts of misfolded SOD1 species to be determined specifically [33, 34]. We have used these methods here to gain information on the effects of various ALS-related pathways on the levels of misfolded SOD1 in patient-specific fibroblasts.

## Materials and Methods

### Human materials

Blood samples and skin biopsies were collected from patients and non-disease controls with approval of the Swedish Ethical Review Board for Medical Research and adhering to the principles of the Declaration of Helsinki (WMA, 1964), following written informed consent (Table A in S1 File).

### *SOD1* and *C9orf72* genotyping

Genomic DNA was extracted from whole blood (buffy coat) using the Nucleon BAAC2 kit (GE Healthcare, Piscataway, NJ, USA) according to the manufacturer's protocol. Amplification of all 5 exons and the corresponding 30–50 bp of flanking intronic regions of the *SOD1* gene was performed using the AmpliTaqGold Kit (Applied Biosystems, Foster City, CA, USA) as described by the manufacturer. Sequencing reactions were performed using BigDye Terminator v3.1 Cycle Sequencing Kit (Applied Biosystems, Foster City, CA, USA) as recommended and analysed using a 3730xl DNA analyzer with the SeqScape Software v2.5 (Applied Biosystems, Foster City, CA, USA). The presence of the GGGGCC-hexanucleotide repeat expansion in *C9orf72* was screened for by repeat-primed polymerase chain reaction (RP-PCR) and fragment length-analysis [35]. Fragment length analysis was performed on a 3730xl DNA analyzer and Peak Scanner Software v1.0 (Applied Biosystems, Foster City, CA, USA). The presence of an expansion was confirmed by Southern blot [35]. All individuals tested negative for mutations in a panel of other ALS-linked genes (details available upon request) and no double mutation carriers were included in the study cohort.

### Cell culture

Following screening of blood to identify *SOD1* and *C9orf72* mutation carriers, a 3 mm punch skin biopsy (upper arm) was obtained from 9 ALS patients with *SOD1* mutations (A4V, H46R, E78_R79insSI, N86S, D90A, G93A, L117V, D125Tfs*24, and G127X), 1 ALS and 1 FTD patient with massive intronic GGGGCC repeat-expansions in *C9orf72*, and 4 non-disease control individuals. The ALS patients were diagnosed according to the EFNS criteria [36]. The G127X biopsy was obtained from an asymptomatic 31 year old carrier of the mutation belonging to a family where 7 other members have developed ALS [5]. All patients were heterozygous for their corresponding mutations except the D90A *SOD1* patient (homozygous). The control subjects were relatives of ALS patients. All healthy control subjects tested negative (wt/wt) for a panel of ALS-associated genes including *SOD1*, *C9orf72*, *TBK1 and UBQLN2*. The study cohort is summarized in Table A in S1 File.

Adult human dermal fibroblast cell lines were established using standard procedures and were maintained in RPMI 1640 medium (Gibco, Life Technologies, Carlsbad, CA, USA), with 25 mM HEPES (Gibco, Life Technologies, Carlsbad, CA, USA), and 2 mM L-glutamine (Gibco, Life Technologies, Carlsbad, CA, USA), 0.1 g/ml penicillin (Meda, Sweden) supplemented with 10% (v/v) foetal bovine serum (Gibco, Life Technologies, Carlsbad, CA, USA) and incubated at 37°C and 5% (v/v) CO_2_.

### Cell treatments

Fibroblasts were plated at a density of around 8000 cells/cm^2^ in 60 mm dishes. After reaching 60–70% confluency, the media were replaced with plain culture media (controls) or media containing the proteasome inhibitor bortezomib (0.5, 5 ng/ml; PS-341, Millennium Pharmaceuticals, USA), 10 mM of the autophagy inhibitor 3-methyladenine (3-MA) (Sigma-Aldrich, St. Louis, MO, USA), 0.5 μg/ml of the glycosylation inhibitor and ER stress inducer tunicamycin (Sigma-Aldrich, St. Louis, MO, USA), and 5 μM of the mitochondrial complex 1 inhibitor rotenone (Sigma-Aldrich, St. Louis, MO, USA). All inhibitor treatments were made in triplicates, with appropriate triplicates of vehicle only controls and the cells were cultured for 24 h before harvest.

### Cell fractionation

Cells were washed with pre-warmed phosphate-buffered saline (PBS; 137 mM NaCl, 2.7 mM KCl, 4.3 mM Na_2_HPO_4_, and 1.4 mM KH_2_PO_4_, pH 7.0) containing 40 mM iodoacetamide (IAM, Thermo Scientific, Rockford, IL, USA), which prevents artificial formation of the C57-C146 disulphide bond in SOD1 by blocking reduced cysteine residues via alkylation [33, 34]. The cells were detached with 0.025% (w/v) trypsin (Gibco, Life Technologies, Carlsbad, CA, USA) and were then washed with PBS containing 40 mM IAM supplemented with 0.5% (v/v) foetal bovine serum to inhibit trypsin and were pelleted at 500 x *g* for 5 min. The cell pellet was resuspended in PBS containing 40 mM IAM and centrifuged at 500 *g* for 5 min. The supernatant was removed and the pellet was snap frozen on dry ice and thereafter kept in a -80°C freezer. For analysis, the cell pellet was rapidly thawed in a 25°C water bath to reduce the effects of cold denaturation. It was then lysed in 500 μl ice-cold PBS containing the Complete EDTA-free protease inhibitor cocktail (Roche Diagnostics, Mannheim, Germany), 40 mM IAM and 0.5% (v/v) NP-40 using a Sonifier Cell Disrupter (Branson, Danbury, CT, USA). The lysate was centrifuged at 20,000 *g* for 30 min at 4°C and the supernatant was collected as the soluble fraction and directly analysed with the misELISA (see below). The resulting pellet was resuspended in ice-cold lysis buffer containing 0.5% (v/v) NP-40 as described above and re-centrifuged twice to remove any contaminating soluble protein. The resulting pellets were used for determination of detergent-insoluble SOD1 aggregates.

### ELISAs for misfolded and total *SOD1*

The ELISA for misfolded SOD1 (misELISA) was carried out as previously detailed [33, 34]. A rabbit antibody raised against a peptide corresponding to amino acids (aa) 24–39 in the human SOD1 sequence was used as primary antibody. This antibody reacts only with disordered SOD1 species and lacks affinity for the natively folded protein [37–39]. A goat anti-human SOD1 antibody was used as secondary antibody. It was raised against SOD1 that had been denatured by incubation with guanidinium chloride and EDTA, and reacts preferentially with the disordered protein [33]. For calibration of the misELISA, a fresh spinal cord from a transgenic mouse expressing G127X mutant human SOD1 was homogenized in 25 volumes 10 mM K phosphate, pH 7.0 in 0.15 M NaCl, containing the Complete anti-proteolytic cocktail (Roche Diagnostics, Mannheim, Germany) and 40 mM IAM. After centrifugation at 20,000 *g*, the supernatant was divided into multiple aliquots that were stored at -80°C. In the misELISA assay, one unit is defined as the amount of “misfolded” SOD1 present in 1 g wet weight of that particular spinal cord.

For ELISA of native/total SOD1, rabbit and goat anti-native human SOD1 antibodies were used [40] following partial purification on Protein A- and Protein G-Sepharose (GE Healthcare Biosciences, Piscataway, NJ, USA), respectively. The rabbit antibody was used as a primary antibody and the goat antibody as a secondary antibody. The ELISA was standardized with a human hemolysate, with the SOD1 content calibrated against pure human SOD1, the concentration of which was determined by quantitative amino acid analysis [40]. The ELISA is able to quantify native SOD1 accurately, but may also respond to some degree to misfolded SOD1 species [33].

### Western blot

The protein concentration of lysates was determined using the BCA Protein Assay Kit (Thermo Scientific, Rockford, IL, USA). Western blots were carried out in Any kD Criterion TGX precast gels (BioRad Laboratories, Hercules, CA, USA) as previously described [7]. The primary antibodies used were; rabbit anti-human SOD1 (0.8 μg/ml, raised against a peptide corresponding to aa 57–72 in the human SOD1) [41], anti-LC3B rabbit (1:1000; Sigma-Aldrich, St. Louis, MO, USA), anti-GRP78 rabbit (1:5000; Novus Biologicals, Littleton, CO, USA), anti-β-actin mouse (1:100 000; Milipore, Bedford, MA, USA). The secondary antibodies were horseradish peroxidase (HRP)-conjugated polyclonal anti-mouse or anti-rabbit IgG (Dako, Glostrup, Denmark). The immunoreaction signal was visualized using an ECL Select reagent (GE Healthcare Biosciences, Piscataway, NJ, USA) recorded on a ChemiDoc apparatus (BioRad Laboratories, Hercules, CA, USA) and analysed using Quantity One software (BioRad Laboratories, Hercules, CA, USA).

### Statistical analyses

Statistical analyses were performed with the Prism 6.0 software (GraphPad, San Diego, CA, USA). For comparison of multiple groups, the one-way ANOVA was used following correction for multiple testing using Tukey’s *post-hoc* analyses. F-test was used to test for equal variance between treatment groups. For *p*-values ≤ 0.05 the Welch test was used. For *p*-values > 0.05 the two-tailed unpaired t-test was used. For comparison of two groups, the Mann-Whitney *U* test was used. The significance level was set to 0.05. All values are given as mean ± SD.

## Results

### Design of experiments

**Fig 1:**
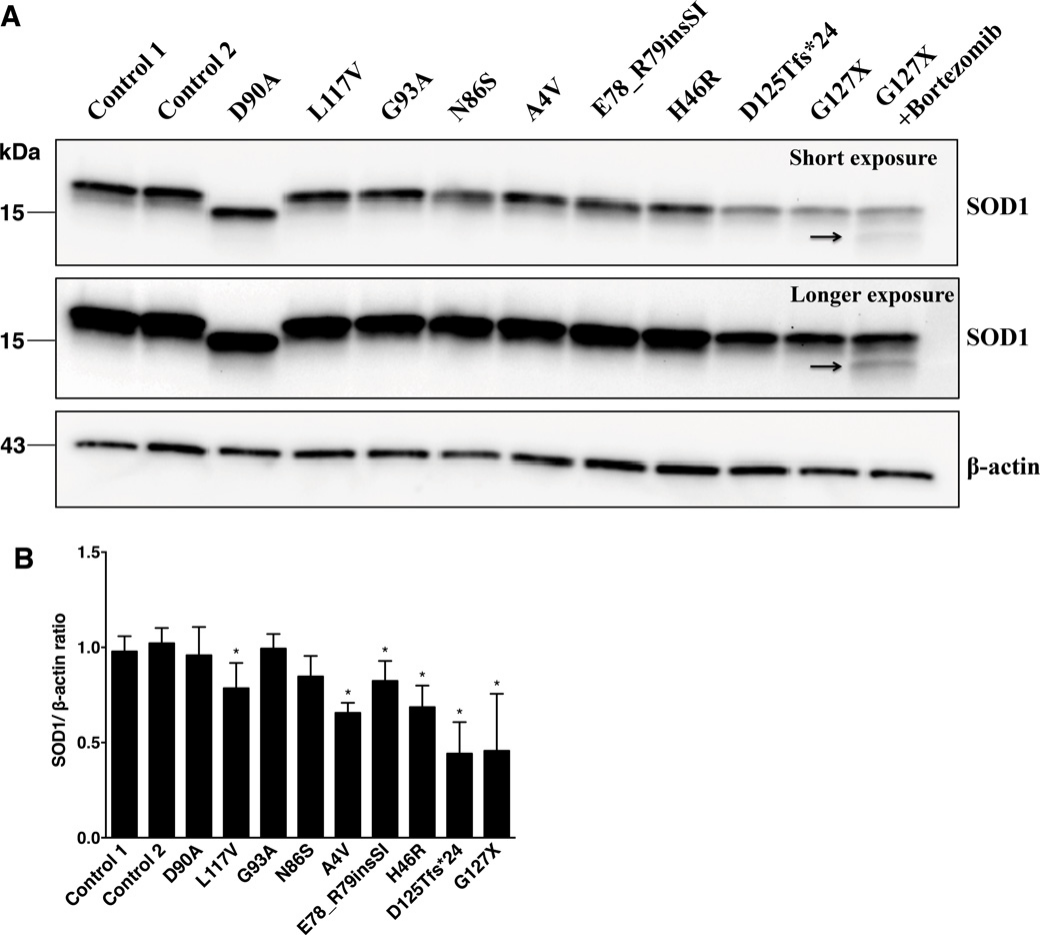
*SOD1* protein in lysates of fibroblast cultures. (A) Fibroblast lysates were analysed by western blotting using the anti-SOD1 (57–72 aa) antibody. β-actin was used as a loading control. The band of the truncated G127X mutant was not visible except in the presence of the proteasome inhibitor bortezomib (arrow). The homozygous D90A mutant displays increased mobility. (B) The expression of SOD1 in the fibroblast lines were compared as SOD1/β-actin ratios. The mean of the 2 control lines was taken as 1. Three replicate sets of fibroblast cultures were analyzed. The data are expressed as means ± SD. (*) p < 0.05 compared to the results for the controls (Mann-Whitney *U* test).

We used fibroblasts derived from patients carrying different *SOD1* mutations and encoding SOD1 variants with widely variable structural characteristics (Table A in S1 File). This approach offered several advantages. Since SOD1 is expressed under the control of the endogenous promoter, treatment responses should better reflect the *in vivo* situation. This also ensures a native, balanced synthesis rate, which when analysed by western blotting resulted in levels of mutant SOD1s ranging from those similar to wild-type SOD1 to non-detectable (Fig 1A). The lysates from fibroblasts expressing the missense mutants D90A, G93A and N86S [9, 42] showed SOD1/β-actin ratios comparable to controls, whereas the ratios were somewhat reduced in lysates from fibroblasts expressing L117V, A4V, E78_R79insSI and H46R (Fig 1B). The disordered, truncated mutants D125Tfs*24 and G127X are rapidly degraded and only SOD1 derived from the wild-type allele was visible. Addition of the proteasome inhibitor bortezomib resulted in low but detectable amounts of the G127X mutant form of SOD1 (Fig 1A).

**Fig 2:**
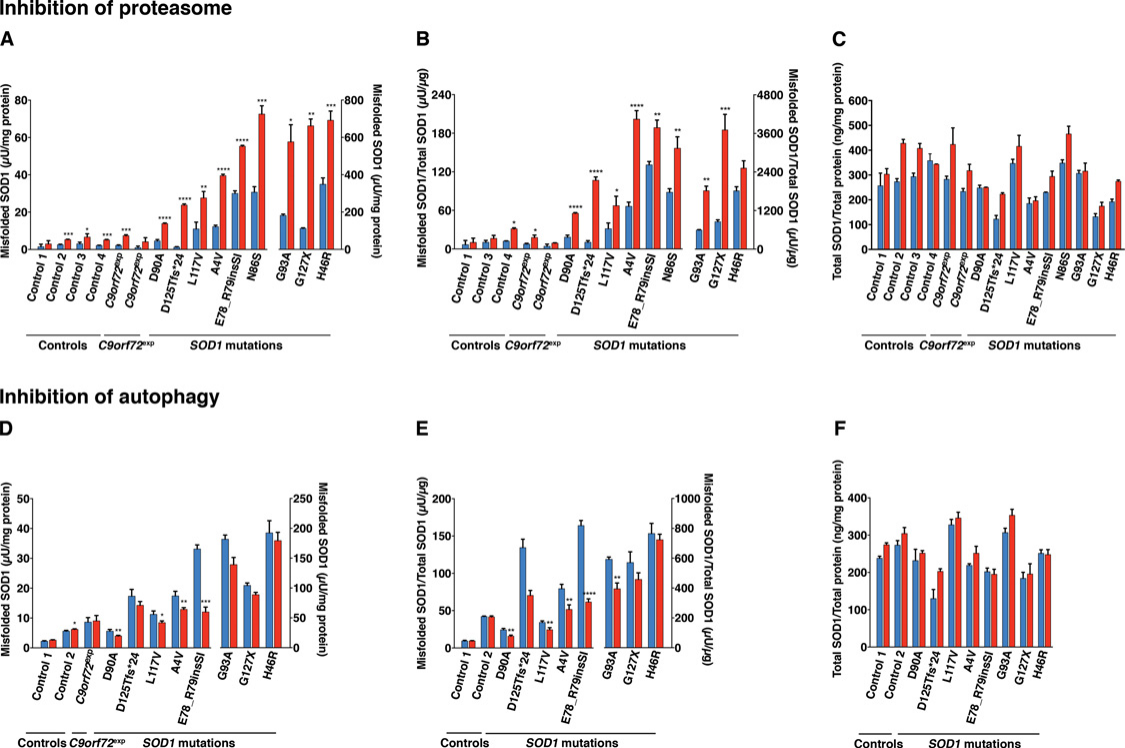
Effect of reduction in the activities of the proteasome and autophagy on the levels of misfolded and total *SOD1* in fibroblast lines. Misfolded and total SOD1 in lysates of fibroblasts were analysed with the 24–39 misELISA and ELISA for total SOD1, respectively. Fibroblast cell lines were cultured in triplicate for 24 h in the absence (blue) or the presence (red) of the proteasome inhibitor bortezomib (A-C; 5 ng/ml), the autophagy inhibitor 3-MA (D-F; 10 mM). Data is expressed as the mean ± SD (n = 3), *p< 0.05, **p< 0.01, ***p< 0.001, ****p< 0.0001 compared to respective non-treated control (Welch test and two-tailed unpaired t-test).

**Fig 3:**
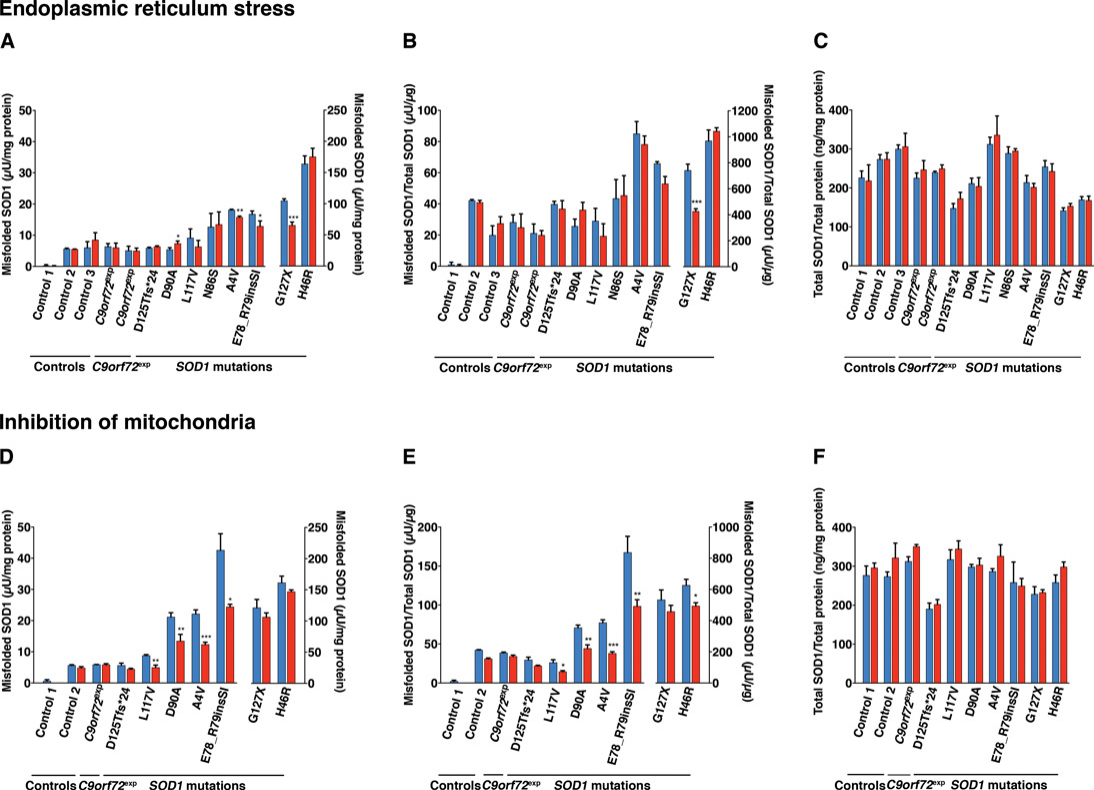
Effect of induction of ER stress and perturbation of mitochondria on the levels of misfolded and total *SOD1* in fibroblast lines. Misfolded and total SOD1 in lysates of fibroblasts were analysed with the 24–39 misELISA and ELISA for total SOD1, respectively. Fibroblast cell lines were cultured in triplicate for 24 h in the absence (blue) and presence (red) of the ER-stress inducer tunicamycin (A-C; 0.5 μg/ml) and the mitochondrial inhibitor rotenone (D-F; 5 μM). Data is expressed as the mean ± SD (n = 3), *p< 0.05, **p< 0.01, ***p< 0.001 compared to respective non-treated control (Welch test and two-tailed unpaired t-test).

We focused primarily on the levels of unfolded/disordered/unstructured/misfolded (in the following we will use the term misfolded) SOD1, since these are the species that are likely to provoke the onset of ALS. We utilized an ELISA, which we have developed and detects misfolded SOD1 species specifically (misELISA) but does not detect holoSOD1, or SOD1 that lacks Cu and/or the C57-C146 disulphide bond, as long as the polypeptide is natively folded [33, 34]. Fibroblast extracts were incubated for 1 h at 23°C with the capture antibody in the misELISAs. The physiological 37°C would have been preferable, but at that temperature some folded SOD1 species have been found to unfold continuously in diluted extracts [34]. Thus, the levels of misfolded SOD1 at physiological temperature are not mirrored and results are not directly comparable between cell lines expressing different SOD1 variants. For a certain fibroblast line, however, the effects of perturbations on the levels of misfolded SOD1 can be determined. The misELISA is very sensitive: note for instance the high readings yielded by the G127X mutant (Figs 2 and 3) which was undetectable by western blot (Fig 1A).

### Effect of protein degradation pathways on misfolded *SOD1* clearance

Degradation by the proteasome is the primary cellular route of misfolded protein elimination [43]. Autophagy plays a fundamental role in the degradation of protein aggregates and also contributes to the elimination of soluble misfolded proteins [43]. Therefore, we determined the effects of inhibition of both systems on misfolded SOD1 levels.

Bortezomib acts primarily as a reversible inhibitor on the chymotrypsin-like site of the 20S proteasome core β5 subunit, but also inhibits the caspase-like site at high concentrations [44]. We first identified a treatment regimen that did not have a significant effect on cell viability, detected using an assay that reports on plasma membrane integrity (Figure A in S1 File). Although we cannot exclude activation of earlier stages of cell death before loss of plasma membrane integrity, under these conditions (5 ng/ml for 24 h) the chymotrypsin-like, caspase-like and trypsin-like activities of the proteasome were inhibited by 96%, 85% and 45%, respectively (Figure B in S1 File). Proteasome inhibition resulted in a marked increase in misfolded SOD1 in all lines tested (Fig 2A and 2B). We also analysed total SOD1 levels in bortezomib-treated cultures. The SOD1/total protein ratios were increased in all, by an average of 28% (Fig 2C). However, expressing the results as misSOD1/total SOD1 ratios did not change the conclusion that proteasome inhibition markedly increased the levels of misfolded SOD1 in the fibroblast cultures (Fig 2B).

We investigated the effects of blocking autophagy using the commonly used inhibitor 3-methyladenine (3-MA) [45]. 3-MA is, however, not a specific inhibitor for autophagy and may affect other cellular processes [46]. Under the current condition -10 mM 3-MA for 24 h- a moderate inhibition was achieved. The levels of microtubule-associated protein light chain 3-II (LC3-II) and the ratio of LC3-II/LC3-I were reduced to 78% and 91%, respectively of controls (Figure C in S1 File). No significant increases in the levels of misfolded SOD1 were detected in any of the lines and in some cases, moderate reductions were recorded (Fig 2D and 2E). Minor increases were seen in total SOD1/total protein ratios in the 3-MA-treated fibroblast cultures (Fig 2F).

### Endoplasmic reticulum stress

ER stress has been observed in post-mortem spinal cord tissue of humans with sporadic ALS [47], transgenic mouse models overexpressing mutant human SOD1 [17–19], cell lines overexpressing mutant SOD1s [17] and A4V iPSCs-derived motor neurons [16]. We sought to investigate the role of ER stress on misfolded SOD1 levels in fibroblasts by exposing them to tunicamycin, an inhibitor of protein glycosylation. Despite strong induction of ER stress measured by the ER chaperone glucose-regulated protein 78 (GRP78) (Figure C in S1 File), no great differences were found in the levels of misSOD1 in any of the cell lines tested (Fig 3A and 3B). ER stress increased the total SOD1/total protein ratios by an average of 3% (Fig 3C).

### Inhibition of mitochondria

Several lines of evidence gathered from studies performed on transgenic mouse and cellular models overexpressing mutant human SOD1 have shown that mutant SOD1s can interfere with mitochondria and this has been suggested to be a primary pathogenic mechanism in ALS [11–14, 16]. Perturbations of mitochondria have also been observed in fibroblasts from a patient carrying a SOD1 mutation [15] and in A4V iPSCs-derived motor neurons [16]. To investigate the effect of mitochondrial inhibition on the level of misfolded SOD1 we used rotenone to inhibit Complex I of the mitochondrial respiratory chain. As shown in Fig 3D and 3E, rotenone treatment caused a moderate reduction in the levels of misfolded SOD1, but no increases. The SOD1/total proteins ratios were increased in all rotenone-treated fibroblast cultures by an average of 7% (Fig 3F).

### Detergent-insoluble aggregates

Accumulation of detergent-insoluble, aggregated forms of mutant SOD1 have been found in motor neurons of ALS patients [25, 28], transgenic mouse models overexpressing mutant human SOD1 [26] as well as cells overexpressing human mutant SOD1s [27, 29, 30] and iPSCs-derived motor neurons [16, 31].

**Fig 4:**
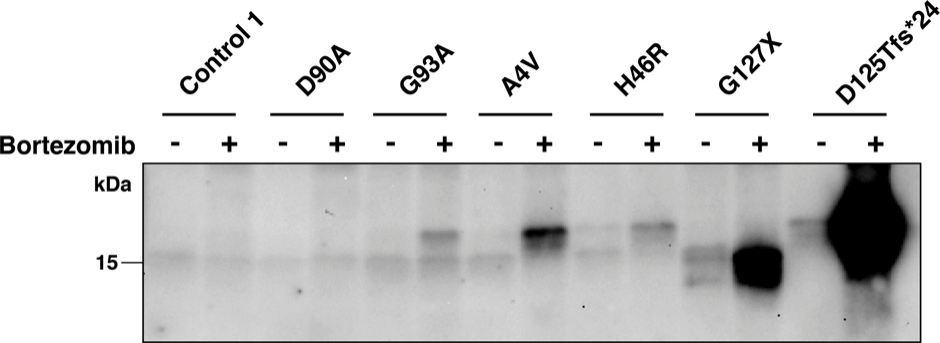
Inhibition of proteasome causes accumulation of detergent-insoluble *SOD1* aggregates. Fibroblast cell lines were cultured in the absence (-) and presence (+) of bortezomib (5 ng/ml) for 24 h. Detergent insoluble fractions were analysed by western blotting using the anti-SOD1 (57–72 aa) antibody. No insoluble aggregates were seen in the control and D90A lines. Proteasome inhibition increased the amount of aggregates in G93A, A4V, H46R, G127X and D125Tfs*24 lines.

We determined the amount of detergent-insoluble aggregates present following all interventions used in study (Fig 4). Despite a high sensitivity of the assay, no aggregates were found in the majority of lines (Table 1). The only untreated cultures that showed detectable aggregates were the cells that expressed H46R, G127X and D125Tfs*24 mutant SOD1s. Moreover, proteasome inhibition was the only intervention that was found to increase aggregate levels. Aggregated species were then also observed in the A4V and G93A lines and there was a remarkable increase in the D125Tfs*24 line (Fig 4, Table 1).

**Table 1:** Detergent-resistant SOD1 aggregates in fibroblast cultures.

	Proteasome inhibition		Autophagy inhibition		ER-stress induction		Inhibition of mitochondria	
	Bortezomib		3-MA		Tunicamycin		Rotenone	
	-	+	-	+	-	+	-	+
**A4V**	<0.03	**0.39**	<0.03	<0.03	<0.03	<0.03	<0.03	<0.03
**H46R**	<0.03	**0.09**	**0.15**	**0.14**	<0.03	<0.03	**0.09**	**0.23**
**N86S**	<0.03	<0.03	n. t.	n. t.	<0.03	<0.03	n. t.	n. t.
**D90A**	<0.03	<0.03	<0.03	<0.03	<0.03	<0.03	<0.03	<0.03
**G93A**	<0.03	**0.04**	<0.03	<0.03	n. t.	n. t.	n. t.	n. t.
**L117V**	<0.03	<0.03	<0.03	<0.03	<0.03	<0.03	<0.03	<0.03
**G127X**	**0.20**	**0.75**	**0.08**	**0.06**	<0.03	<0.03	<0.03	<0.03
**E78_R79insSI**	<0.03	<0.03	<0.03	<0.03	<0.03	<0.03	<0.03	<0.03
D125Tfs*24	**0.22**	**25.26**	**0.49**	**0.24**	**0.73**	**0.41**	**0.35**	**0.30**
***C9orf72*^exp^**	<0.03	<0.03	<0.03	<0.03	<0.03	<0.03	<0.03	<0.03
***C9orf72*^exp^**	<0.03	<0.03	n. t.	n. t.	<0.03	<0.03	n. t.	n. t.
**Control**	<0.03	<0.03	<0.03	<0.03	<0.03	<0.03	<0.03	<0.03

The detergent-resistant aggregates were quantified from western blots. Amounts are presented as % of total amounts of SOD1 in the cultures as measured by ELISA. The detection limit for SOD1 aggregates in the cultures was approximately 0.03% of total SOD1, n. t. = not tested.

In the case of aggregates detected in fibroblasts bearing A4V, H46R and G93A mutations, we were unable to distinguish between the mutant protein and wild-type SOD1. However, we could confirm aggregation of the truncated G127X and D125Tfs*24 mutants by western blotting using an anti-SOD1 antibody raised against a peptide in the C-terminal end (amino acids 144–153), which is lacking in these mutants. Using this antibody, which only would detect wild-type SOD1, no aggregates were detectable (Figure D in S1 File).

### Fibroblasts from patients carrying *C9orf72* expansions

Using antibodies specific for misfolded SOD1 species, we have previously shown that SOD1-immunoreactive inclusions can occur in motor neurons and glia in patients with sporadic as well as familial ALS without *SOD1* mutations [37, 38]. Therefore, we analysed misSOD1 in fibroblasts from patients carrying the GGGGCC-repeat-expansion in *C9orf72*. The misSOD1 levels, as well as the changes induced by the various treatments, did not differ between the non-disease control cultures and the fibroblast cultures derived from patients carrying *C9orf72* expansions (Figs 2 and 3).

## Discussion

Cellular models of ALS have been used widely to investigate disease-related pathological abnormalities. These have been mostly based on overexpression of mutant human SOD1s in various cell types [27, 29, 30]. In this study we have performed the first comprehensive analysis of misfolded, aggregated and total SOD1 in a variety of fibroblast cell lines obtained from ALS patients carrying mutations in *SOD1*, as well as from patients carrying the GGGGCC-repeat-expansion in *C9orf72* and from non-disease controls. The major advantage of patient-derived fibroblasts is that they express SOD1s under the control of the endogenous promoter ensuring an appropriately regulated and physiological level of SOD1 expression. We took advantage of these cells to gain insight into the effects interfering with various ALS-related pathways on the levels of misfolded SOD1 as analysed by misELISA.

We found elevated levels of misfolded SOD1 in fibroblast lines that expressed mutant SOD1 compared to the non-disease controls. ER stress has been shown to be enhanced in patient-derived motor neurons carrying a *SOD1* A4V mutation [16]. However, following a 24 h treatment with tunicamycin, ER stress did not result in any significant changes in misfolded SOD1 levels in fibroblasts despite a robust increase in GRP78 levels. We cannot exclude that sustained ER stress might lead to an increase in misfolded SOD1, or that mitotically active fibroblasts show a different response to terminally differentiated motor neurons. Inhibition of mitochondria and autophagy in some cases lead to reduced levels of misfolded SOD1, however, we did not observe a consistent pattern to this and it might be explained by a differential response of cell lines to the treatments and perhaps reduced protein and SOD1 synthesis. The only perturbation that consistently and uniformly increased the levels of misfolded SOD1 was proteasome inhibition. Clearly these increases can not be explained only by increased levels of total mutant SOD1s: misfolded D90A SOD1 rose 3-fold, but no significant change in total mutant protein was observed in fibroblasts from the homozygous D90A patient. It is also notable that, although the levels were low, we observed increases in misfolded SOD1 in the non-disease control fibroblast cultures (average of 2.1 fold). In this context it is of interest to note that inclusions containing misfolded SOD1 also have been found in motor neurons from ALS patients lacking *SOD1* mutations [37, 38, 48]. Several previous studies have reported that proteasome inhibition can increase total levels of overexpressed mutant SOD1s, but amounts of misfolded SOD1 species have not been specifically analysed [29, 49]. Autophagy inhibition has also been reported to increase levels of total mutant SOD1 in overexpressing cells [29].

Inclusions containing aggregated SOD1 are a hallmark of ALS, both in patients at end stage and in transgenic animal models overexpressing mutant SOD1 [26, 28]. Therefore, we investigated the appearance of detergent-resistant aggregates in fibroblasts. In most cases none were found. The only lines that were found to contain aggregates were those expressing the truncated SOD1 variants D125Tfs*24 and G127X, and the missense variant H46R. Proteasome inhibition increased the levels of soluble misfolded and detergent-resistant aggregates of SOD1 in these, and caused the appearance of SOD1 aggregates in the A4V, H46R and G93A cell lines. The levels were generally very low; below 1% of the total SOD1 present in the lysates. This is different from findings in overexpressed cell culture systems where 10-fold larger proportions are typically found as aggregated species [30]. SOD1 is able to form several different strains of aggregate *in vivo* and *in vitro* [39]. It is not yet known whether or not SOD1 aggregates formed in cell culture bear any relation to those that arise and propagate *in vivo*, however, our results show that patient-derived fibroblasts could be a useful model with which to study the aggregation process.

The D125Tfs*24 mutant form of SOD1 stands out as remarkable among the mutants studied here. This mutation leads to a frameshift and the inclusion of a 23 aa long neopeptide followed by a stop codon (Table A in S1 File) [50]. Under basal conditions, the levels of misfolded SOD1 detected by the misELISA are low and comparable with those of fibroblast cultures without *SOD1* mutations, yet specific aggregates of the mutant protein were detected (Fig 4). Following proteasome inhibition the relative increase in misfolded SOD1 levels were found to be the greatest among the mutants tested, and copious amounts of mutant SOD1 aggregates were formed. Apparently, this mutant is efficiently targeted for proteasome degradation, which perhaps compensates for its huge aggregation propensity since this *SOD1* mutation is associated with a very slowly progressing disease phenotype (D. Lange, unpublished data).

We show that patient-derived fibroblasts, with physiological levels of SOD1 expression, recapitulate some of the aspects of mutant SOD1 that are known to be important for pathophysiology of ALS, namely increased levels of misfolded SOD1 and presence of detergent insoluble aggregates. Despite the fact that these cells are mitotically active and may not accurately mimic the cellular environment of aged, post-mitotic motor neurons, these properties make them a useful model to study ALS-related cellular pathway disturbances.

In conclusion, we have found, somewhat surprisingly, that several of the disturbances associated with SOD1-provoked motor neuron disease including mitochondrial dysfunction and ER stress do not increase SOD1 misfolding or aggregation *per se*. Nor does autophagy inhibition, however, we hesitate to rule out a role in aggregate degradation since the degree of inhibition achieved was moderate. The ubiquitin-proteasome system has been found to be inhibited in mutant SOD1-expressing ALS-model mice [21, 22]. Together with autophagy, the activity of the ubiquitin-proteasome pathway has also been found to decline with aging [51]. Thus, proteasome impairment with age could be one of the key factors behind the age-related increase in the incidence of ALS and maybe in forming an amplifying vicious circle that leads to misfolded SOD1 accumulation.

## Supporting Information

S1 File Supporting Materials and Methods, Figures, and Table. Measurement of in vitro cell cytotoxicity in the fibroblast lines following proteasome inhibition. (Figure A). Inhibition of chymotrypsin-like (A), caspase-like (B) and trypsin-like (C) proteasome activities in the fibroblast lines following 0.5 and 5 ng/ml bortezomib treatment. (Figure B). Determination of the efficacy of 3-MA and tunicamycin treatments. Western blots showing the relative amounts of (A) LC3-I and LC3-II and (B) GRP78. (Figure C). Detection of full length SOD1 in detergent-resistant aggregates in fibroblasts. (Figure D). Study cohort. (Table A).
(DOCX)

## References

[1] Charcot JM. Lecons sur les maladies du système nervaux. 1873. p. 163–204.

[2] Rosen DR, Siddique T, Patterson D, Figlewicz DA, Sapp P, Hentati A, et al Mutations in Cu/Zn superoxide dismutase gene are associated with familial amyotrophic lateral sclerosis. Nature. 1993;362:59–62. 10.1038/362059a0 . 8446170

[3] Andersen PM, Al-Chalabi A. Clinical genetics of amyotrophic lateral sclerosis: what do we really know? Nat Rev Neurol. 2011;7:603–15. 10.1038/nrneurol.2011.150 . 21989245

[4] Wroe R, Wai-Ling Butler A, Andersen PM, Powell JF, Al-Chalabi A. ALSOD: the Amyotrophic Lateral Sclerosis Online Database. Amyotroph Lateral Scler. 2008;9:249–50. 10.1080/17482960802146106 . 18608099

[5] Andersen PM, Nilsson P, Keränen ML, Forsgren L, Hägglund J, Karlsborg M, et al Phenotypic heterogeneity in motor neuron disease patients with CuZn-superoxide dismutase mutations in Scandinavia. Brain. 1997;120:1723–37. . 9365366 10.1093/brain/120.10.1723

[6] Andersen PM, Sims KB, Xin WW, Kiely R, O'Neill G, Ravits J, et al Sixteen novel mutations in the Cu/Zn superoxide dismutase gene in amyotrophic lateral sclerosis: a decade of discoveries, defects and disputes. Amyotroph Lateral Scler Other Motor Neuron Disord. 2003;4:62–73. . 14506936 10.1080/14660820310011700

[7] Jonsson PA, Ernhill K, Andersen PM, Bergemalm D, Brännström T, Gredal O, et al Minute quantities of misfolded mutant superoxide dismutase-1 cause amyotrophic lateral sclerosis. Brain. 2004;127:73–88. 10.1093/brain/awh005 . 14534160

[8] Andersen PM, Nilsson P, Ala-Hurula V, Keränen ML, Tarvainen I, Haltia T, et al Amyotrophic lateral sclerosis associated with homozygosity for an Asp90Ala mutation in CuZn-superoxide dismutase. Nat Genet. 1995;10:61–6. 10.1038/ng0595-61 . 7647793

[9] Synofzik M, Ronchi D, Keskin I, Basak AN, Wilhelm C, Gobbi C, et al Mutant superoxide dismutase-1 indistinguishable from wild-type causes ALS. Hum Mol Genet. 2012;21:3568–74. 10.1093/hmg/dds188 . 22595972

[10] Sato T, Nakanishi T, Yamamoto Y, Andersen PM, Ogawa Y, Fukada K, et al Rapid disease progression correlates with instability of mutant SOD1 in familial ALS. Neurology. 2005;65:1954–7. 10.1212/01.wnl.0000188760.53922.05 . 16291929

[11] Menzies FM, Cookson MR, Taylor RW, Turnbull DM, Chrzanowska-Lightowlers ZM, Dong L, et al Mitochondrial dysfunction in a cell culture model of familial amyotrophic lateral sclerosis. Brain. 2002;125:1522–33. . 12077002 10.1093/brain/awf167

[12] Mattiazzi M, D'Aurelio M, Gajewski CD, Martushova K, Kiaei M, Beal MF, et al Mutated human SOD1 causes dysfunction of oxidative phosphorylation in mitochondria of transgenic mice. J Biol Chem. 2002;277:29626–33. 10.1074/jbc.M203065200 . 12050154

[13] Israelson A, Arbel N, Da Cruz S, Ilieva H, Yamanaka K, Shoshan-Barmatz V, et al Misfolded mutant SOD1 directly inhibits VDAC1 conductance in a mouse model of inherited ALS. Neuron. 2010;67:575–87. 10.1016/j.neuron.2010.07.019 . 20797535 PMC2941987

[14] Pickles S, Destroismaisons L, Peyrard SL, Cadot S, Rouleau GA, Brown RH, et al Mitochondrial damage revealed by immunoselection for ALS-linked misfolded SOD1. Hum Mol Genet. 2013;22:3947–59. 10.1093/hmg/ddt249 . 23736301 PMC5052069

[15] Allen SP, Rajan S, Duffy L, Mortiboys H, Higginbottom A, Grierson AJ, et al Superoxide dismutase 1 mutation in a cellular model of amyotrophic lateral sclerosis shifts energy generation from oxidative phosphorylation to glycolysis. Neurobiol Aging. 2014;35:1499–509. 10.1016/j.neurobiolaging.2013.11.025 . 24439480

[16] Kiskinis E, Sandoe J, Williams LA, Boulting GL, Moccia R, Wainger BJ, et al Pathways disrupted in human ALS motor neurons identified through genetic correction of mutant SOD1. Cell Stem Cell. 2014;14:781–95. 10.1016/j.stem.2014.03.004 . 24704492 PMC4653065

[17] Atkin JD, Farg MA, Turner BJ, Tomas D, Lysaght JA, Nunan J, et al Induction of the unfolded protein response in familial amyotrophic lateral sclerosis and association of protein-disulfide isomerase with superoxide dismutase 1. J Biol Chem. 2006;281:30152–65. 10.1074/jbc.M603393200 . 16847061

[18] Kikuchi H, Almer G, Yamashita S, Guégan C, Nagai M, Xu Z, et al Spinal cord endoplasmic reticulum stress associated with a microsomal accumulation of mutant superoxide dismutase-1 in an ALS model. Proc Natl Acad Sci U S A. 2006;103:6025–30. 10.1073/pnas.0509227103 . 16595634 PMC1458691

[19] Saxena S, Cabuy E, Caroni P. A role for motoneuron subtype-selective ER stress in disease manifestations of FALS mice. Nat Neurosci. 2009;12:627–36. 10.1038/nn.2297 . 19330001

[20] Urushitani M, Kurisu J, Tsukita K, Takahashi R. Proteasomal inhibition by misfolded mutant superoxide dismutase 1 induces selective motor neuron death in familial amyotrophic lateral sclerosis. J Neurochem. 2002;83:1030–42. . 12437574 10.1046/j.1471-4159.2002.01211.x

[21] Cheroni C, Marino M, Tortarolo M, Veglianese P, De Biasi S, Fontana E, et al Functional alterations of the ubiquitin-proteasome system in motor neurons of a mouse model of familial amyotrophic lateral sclerosis. Hum Mol Genet. 2009;18:82–96. 10.1093/hmg/ddn319 . 18826962 PMC3298865

[22] Kabashi E, Agar JN, Strong MJ, Durham HD. Impaired proteasome function in sporadic amyotrophic lateral sclerosis. Amyotroph Lateral Scler. 2012;13:367–71. 10.3109/17482968.2012.686511 . 22632443

[23] Zhang F, Ström AL, Fukada K, Lee S, Hayward LJ, Zhu H. Interaction between familial amyotrophic lateral sclerosis (ALS)-linked SOD1 mutants and the dynein complex. J Biol Chem. 2007;282:16691–9. 10.1074/jbc.M609743200 . 17403682

[24] Li L, Zhang X, Le W. Altered macroautophagy in the spinal cord of SOD1 mutant mice. Autophagy. 2008;4:290–3. . 18196963 10.4161/auto.5524

[25] Shibata N, Hirano A, Kobayashi M, Siddique T, Deng HX, Hung WY, et al Intense superoxide dismutase-1 immunoreactivity in intracytoplasmic hyaline inclusions of familial amyotrophic lateral sclerosis with posterior column involvement. J Neuropathol Exp Neurol. 1996;55:481–90. . 8786408 10.1097/00005072-199604000-00011

[26] Bruijn LI, Becher MW, Lee MK, Anderson KL, Jenkins NA, Copeland NG, et al ALS-linked SOD1 mutant G85R mediates damage to astrocytes and promotes rapidly progressive disease with SOD1-containing inclusions. Neuron. 1997;18:327–38. . 9052802 10.1016/s0896-6273(00)80272-x

[27] Durham HD, Roy J, Dong L, Figlewicz DA. Aggregation of mutant Cu/Zn superoxide dismutase proteins in a culture model of ALS. J Neuropathol Exp Neurol. 1997;56:523–30. . 9143265 10.1097/00005072-199705000-00008

[28] Kato S, Takikawa M, Nakashima K, Hirano A, Cleveland DW, Kusaka H, et al New consensus research on neuropathological aspects of familial amyotrophic lateral sclerosis with superoxide dismutase 1 (SOD1) gene mutations: inclusions containing SOD1 in neurons and astrocytes. Amyotroph Lateral Scler Other Motor Neuron Disord. 2000;1:163–84. . 11464950 10.1080/14660820050515160

[29] Kabuta T, Suzuki Y, Wada K. Degradation of amyotrophic lateral sclerosis-linked mutant Cu,Zn-superoxide dismutase proteins by macroautophagy and the proteasome. J. Biol. Chem. 2006 p. 30524–33. 16920710 10.1074/jbc.M603337200

[30] Prudencio M, Hart PJ, Borchelt DR, Andersen PM. Variation in aggregation propensities among ALS-associated variants of SOD1: correlation to human disease. Hum Mol Genet. 2009;18:3217–26. 10.1093/hmg/ddp260 . 19483195 PMC2722984

[31] Chen H, Qian K, Du Z, Cao J, Petersen A, Liu H, et al Modeling ALS with iPSCs reveals that mutant SOD1 misregulates neurofilament balance in motor neurons. Cell Stem Cell. 2014;14:796–809. 10.1016/j.stem.2014.02.004 . 24704493 PMC4230530

[32] Synofzik M, Fernández-Santiago R, Maetzler W, Schöls L, Andersen PM. The human G93A SOD1 phenotype closely resembles sporadic amyotrophic lateral sclerosis. J Neurol Neurosurg Psychiatry. 2010;81:764–7. 10.1136/jnnp.2009.181719 . 20176600

[33] Zetterström P, Andersen PM, Brännström T, Marklund SL. Misfolded superoxide dismutase-1 in CSF from amyotrophic lateral sclerosis patients. J Neurochem. 2011;117:91–9. 10.1111/j.1471-4159.2011.07177.x . 21226712

[34] Zetterström P, Graffmo KS, Andersen PM, Brännström T, Marklund SL. Composition of soluble misfolded superoxide dismutase-1 in murine models of amyotrophic lateral sclerosis. Neuromolecular Med. 2013;15:147–58. 10.1007/s12017-012-8204-z . 23076707

[35] Akimoto C, Volk AE, van Blitterswijk M, Van den Broeck M, Leblond CS, Lumbroso S, et al A blinded international study on the reliability of genetic testing for GGGGCC-repeat expansions in C9orf72 reveals marked differences in results among 14 laboratories. J Med Genet. 2014;51:419–24. 10.1136/jmedgenet-2014-102360 . 24706941 PMC4033024

[36] Andersen PM, Borasio GD, Dengler R, Hardiman O, Kollewe K, Leigh PN, et al EFNS task force on management of amyotrophic lateral sclerosis: guidelines for diagnosing and clinical care of patients and relatives. Eur J Neurol. 2005;12:921–38. 10.1111/j.1468-1331.2005.01351.x . 16324086

[37] Forsberg K, Jonsson PA, Andersen PM, Bergemalm D, Graffmo KS, Hultdin M, et al Novel antibodies reveal inclusions containing non-native SOD1 in sporadic ALS patients. PLoS One. 2010;5:e11552 10.1371/journal.pone.0011552 . 20644736 PMC2904380

[38] Forsberg K, Andersen PM, Marklund SL, Brännström T. Glial nuclear aggregates of superoxide dismutase-1 are regularly present in patients with amyotrophic lateral sclerosis. Acta Neuropathol. 2011;121:623–34. 10.1007/s00401-011-0805-3 . 21287393 PMC3085063

[39] Bergh J, Zetterström P, Andersen PM, Brännström T, Graffmo KS, Jonsson PA, et al Structural and kinetic analysis of protein-aggregate strains in vivo using binary epitope mapping. Proc Natl Acad Sci U S A. 2015;112:4489–94. 10.1073/pnas.1419228112 . 25802384 PMC4394267

[40] Marklund SL, Andersen PM, Forsgren L, Nilsson P, Ohlsson PI, Wikander G, et al Normal binding and reactivity of copper in mutant superoxide dismutase isolated from amyotrophic lateral sclerosis patients. J Neurochem. 1997;69:675–81. . 9231727 10.1046/j.1471-4159.1997.69020675.x

[41] Jonsson PA, Graffmo KS, Andersen PM, Brännström T, Lindberg M, Oliveberg M, et al Disulphide-reduced superoxide dismutase-1 in CNS of transgenic amyotrophic lateral sclerosis models. Brain. 2006;129:451–64. 10.1093/brain/awh704 . 16330499

[42] Jonsson PA, Graffmo KS, Brännström T, Nilsson P, Andersen PM, Marklund SL. Motor neuron disease in mice expressing the wild type-like D90A mutant superoxide dismutase-1. J Neuropathol Exp Neurol. 2006;65:1126–36. 10.1097/01.jnen.0000248545.36046.3c . 17146286

[43] Ciechanover A, Kwon YT. Degradation of misfolded proteins in neurodegenerative diseases: therapeutic targets and strategies. Exp Mol Med. 2015;47:e147 10.1038/emm.2014.117 . 25766616 PMC4351408

[44] Paramore A, Frantz S. Bortezomib. Nat Rev Drug Discov. 2003;2:611–2. 10.1038/nrd1159 . 12908468

[45] Seglen PO, Gordon PB. 3-Methyladenine: specific inhibitor of autophagic/lysosomal protein degradation in isolated rat hepatocytes. Proc Natl Acad Sci U S A. 1982;79:1889–92. . 6952238 10.1073/pnas.79.6.1889 PMC346086

[46] Mizushima N, Yoshimori T, Levine B. Methods in mammalian autophagy research. Cell. 2010;140:313–26. 10.1016/j.cell.2010.01.028 . 20144757 PMC2852113

[47] Ilieva EV, Ayala V, Jové M, Dalfó E, Cacabelos D, Povedano M, et al Oxidative and endoplasmic reticulum stress interplay in sporadic amyotrophic lateral sclerosis. Brain. 2007;130:3111–23. 10.1093/brain/awm190 . 17716997

[48] Bosco DA, Morfini G, Karabacak NM, Song Y, Gros-Louis F, Pasinelli P, et al Wild-type and mutant SOD1 share an aberrant conformation and a common pathogenic pathway in ALS. Nat Neurosci. 2010;13:1396–403. 10.1038/nn.2660 . 20953194 PMC2967729

[49] Hetz C, Thielen P, Matus S, Nassif M, Court F, Kiffin R, et al XBP-1 deficiency in the nervous system protects against amyotrophic lateral sclerosis by increasing autophagy. Genes Dev. 2009;23:2294–306. 10.1101/gad.1830709 . 19762508 PMC2758741

[50] Brown JA, Min J, Staropoli JF, Collin E, Bi S, Feng X, et al SOD1, ANG, TARDBP and FUS mutations in amyotrophic lateral sclerosis: a United States clinical testing lab experience. Amyotroph Lateral Scler. 2012;13:217–22. 10.3109/17482968.2011.643899 . 22292843

[51] Bishop NA, Lu T, Yankner BA. Neural mechanisms of ageing and cognitive decline. Nature. 2010;464:529–35. 10.1038/nature08983 . 20336135 PMC2927852

