# Supplementary material for: Effects of Cellular Pathway Disturbances on Misfolded Superoxide Dismutase-1 in Fibroblasts Derived from ALS Patients
Source: PLoS One. 2016 Feb 26;11(2):e0150133. doi: 10.1371/journal.pone.0150133 (PMC4769150; doi:10.1371/journal.pone.0150133)
Supplement: S1 File — Measurement of in vitro cell cytotoxicity in the fibroblast lines following proteasome inhibition. (Figure A). Inhibition of chymotrypsin-like (A), caspase-like (B) and trypsin-like (C) proteasome activities in the fibroblast lines following 0.5 and 5 ng/ml bortezomib treatment. (Figure B). Determination of the efficacy of 3-MA and tunicamycin treatments. Western blots showing the relative amounts of (A) LC3-I and LC3-II and (B) GRP78. (Figure C). Detection of full length SOD1 in detergent-resistant aggregates in fibroblasts. (Figure D). Study cohort. (Table A). (DOCX) [file pone.0150133.s001.docx]

**Supporting Information**

**Effects of cellular pathway disturbances on misfolded superoxide dismutase-1 in fibroblasts derived from ALS patients**

Keskin et al.

**Supporting Materials and Methods**

**In vitro cell cytotoxicity assay**

Cells were plated at a density of 8000 cells/cm^2^ in 96-well plates (µClear, black-walled; Greiner Bio-One, Frickenhausen, Germany) and incubated overnight. They were then treated with bortezomib (0.05, 0.15, 0.5, 1.5, 5 and 15 ng/ml) or media alone for 24 h at 37°C. A luminescent cytotoxicity assay (CytoTox-Glo™, Promega, Madison, WI, USA) was used in order to establish the optimal concentration of bortezomib for proteasome inhibition without inducing cytotoxicity. This assay detects protease activity in dead cells that have lost plasma membrane integrity using the luminogenic substrate AAF-Glo™. Total cell number was quantified after the addition of digitonin to permeabilise the plasma membrane. Cell viability was calculated by subtracting the luminescence signal obtained before permeabilization from the total luminescence values obtained afterwards. Analyses were performed according to the manufacturer’s protocol using a Synergy 2 multi-mode microplate reader (Biotek, Winooski, VT, USA). All treatments were performed in triplicate and the mean ± SD was determined.

**Proteasome activity assay**

Cells were plated at a density of 8000 cells/cm^2^ in 96-well plates (µClear, black-walled; Greiner Bio-One, Frickenhausen, Germany) and incubated overnight. They were then exposed to bortezomib (0.5 and 5 ng/ml) or media alone for 24 h at 37°C. The proteolytic activities of the proteasome were assayed using the Cell-Based Proteasome-Glo™ Assay kit (Promega, Madison, WI, USA). The assay includes the substrates Suc-LLVY-aminoluciferin for chymotrypsin-like, Z-LRR-aminoluciferin for trypsin-like, and Z-nLPnLD-aminoluciferin for caspase-like activities. The assay was performed according to the manufacturer’s protocol and analysed using a Synergy 2 multi-mode microplate reader (Biotek, Winooski, VT, USA). All treatments were performed in triplicate and the mean ± SD is presented.

**Supporting Figures**

**

**

**Figure A. Measurement of *in vitro* cell cytotoxicity in the fibroblast lines following proteasome inhibition.**

Bar chart showing the relative number of dead (A), total (B) and live (C) cells following treatment with different concentration of bortezomib. Results obtained using the AAF-Glo reagent are given as the mean ± SD (n=3) (one-way ANOVA and Tukey’s *post-hoc* test).

**
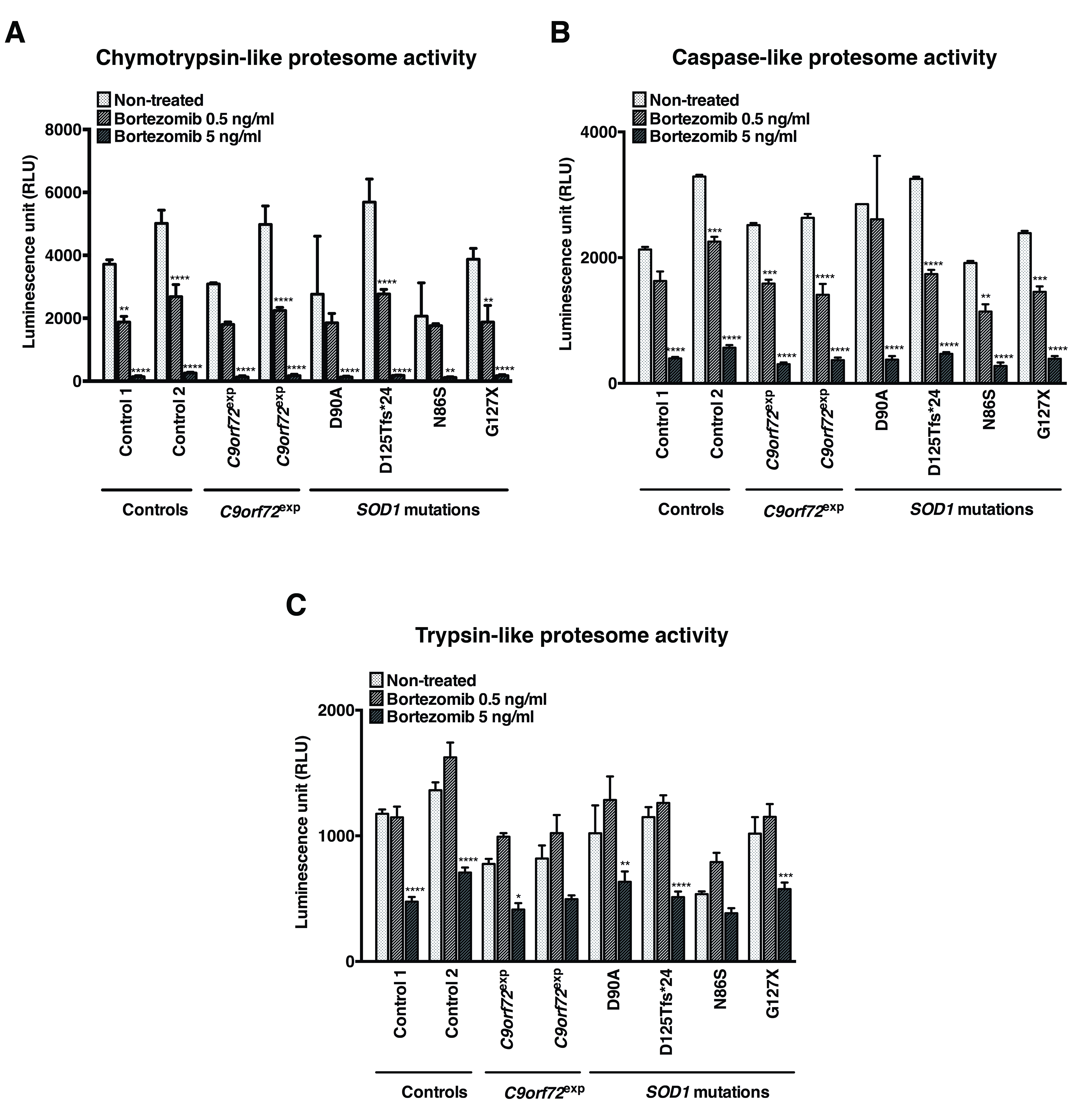
**

**Figure B.** **Inhibition of chymotrypsin-like (A), caspase-like (B) and trypsin-like (C) proteasome activities in the fibroblast lines following 0.5 and 5 ng/ml bortezomib treatment.**

Results are given as the mean ± SD (n=3), **p*< 0.05, ***p*< 0.01, ****p*< 0.001, *****p*< 0.0001 compared to respective non-treated control (one-way ANOVA and Tukey’s *post-hoc* test).


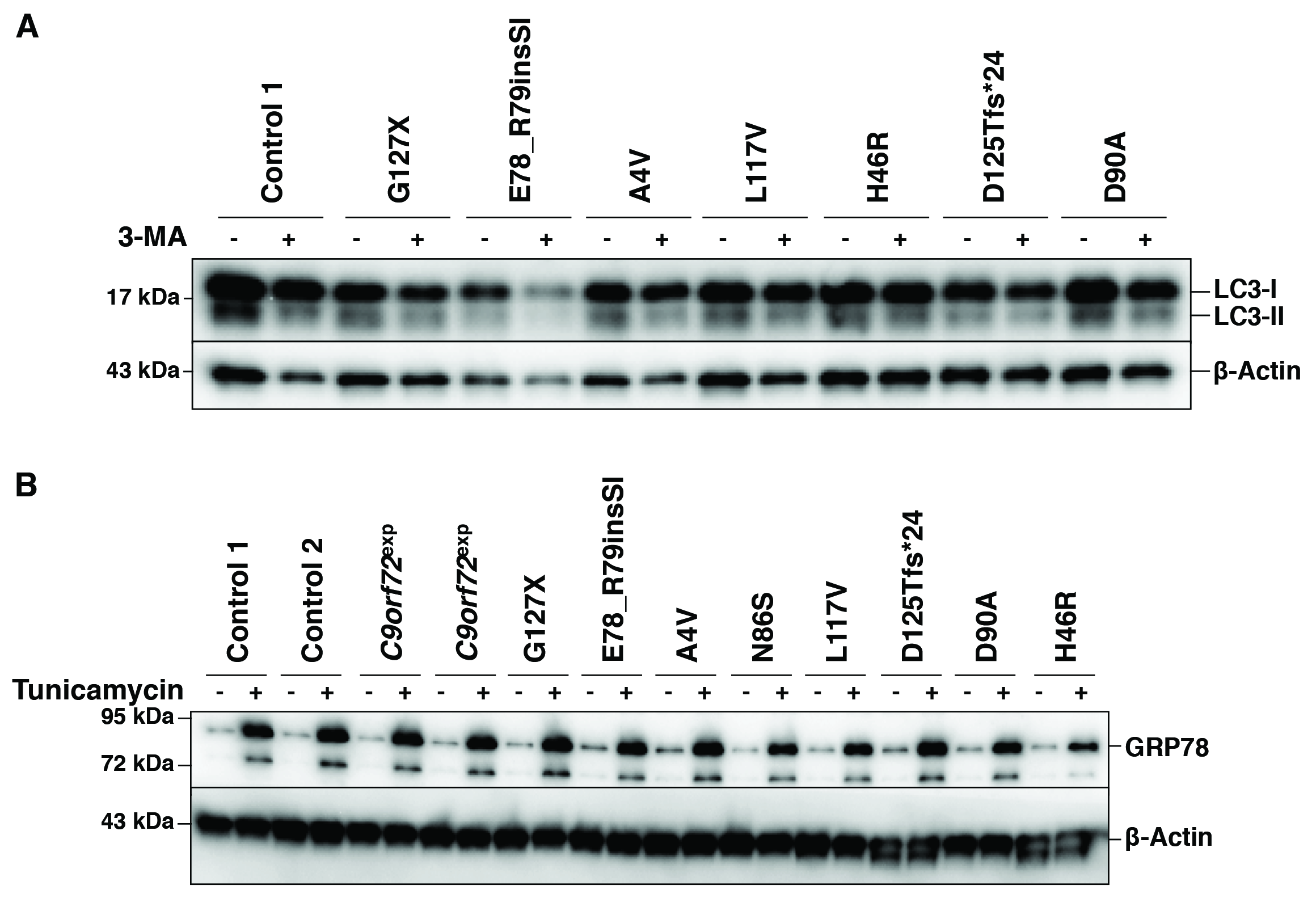


**Figure C.** **Determination of the efficacy of 3-MA and tunicamycin treatments. Western blots showing the relative amounts of (A) LC3-I and LC3-II and (B) GRP78.**

A subset of the fibroblast lines were treated in the absence (-) and presence (+) of 10 mM 3-MA for autophagy inhibition and 0.5 µg/ml tunicamycin for induction of ER stress for 24 h and total cell lysates were analysed by western blotting. β-actin was used as a loading control. The relative level of proteins was quantified by densitometry.


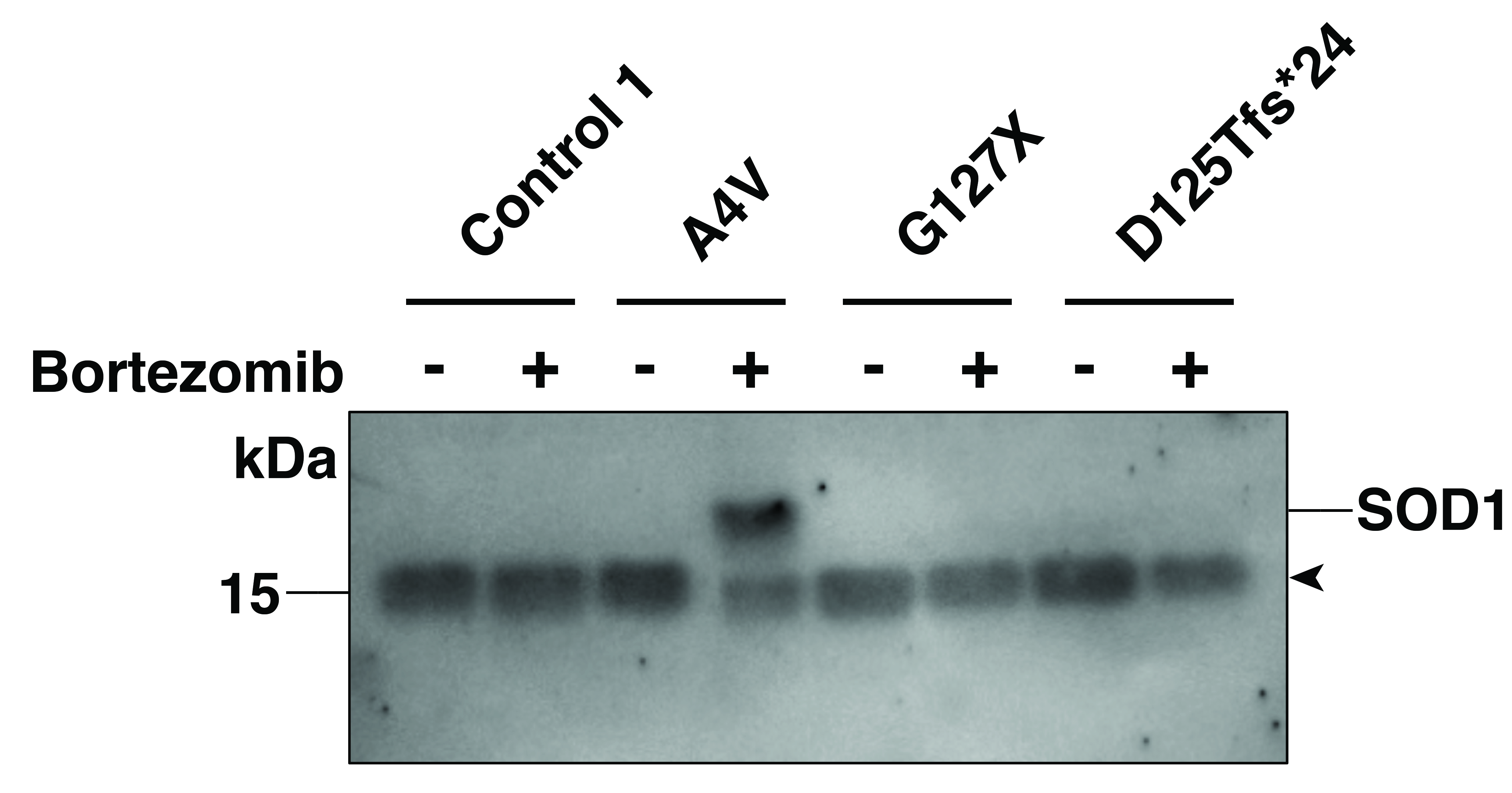


**Figure D.** **Detection of full length SOD1 in** **detergent-resistant aggregates in fibroblasts.**

Fibroblast cell lines were cultured in the absence (-) and presence (+) of bortezomib (5 ng/ml) for 24 h. Aggregation of the truncated G127X and D125Tfs*24 mutants alone was confirmed by western blotting using an anti-SOD1 antibody raised against a peptide in the C-terminal end (144-153 aa), which is lacking in these mutants (*cf.* Fig 4 also). Using this antibody, which only reacts with full-length wild-type SOD1, no aggregates were detectable. Note cross reactivity with a 15 kDa species using this anti-SOD1 antibody (arrowhead).

**Supporting Table**

**Table A. Study cohort.**

| Nr | Diagnosis | Genotype | Sex | Age  onset  (years) | Survival  time  (months) | Year  biopsy obtained | SOD1 activity U/mg Hb*** | Comments |
| --- | --- | --- | --- | --- | --- | --- | --- | --- |
| 1 | Control 1 | wt/wt | f | n a | n a | 2009 | 57 |  |
| 2 | Control 2 | wt/wt | f | n a | n a | 2014 | 56 |  |
| 3 | Control 3 | wt/wt | f | n a | n a | 2007 | 53 |  |
| 4 | Control 4 | wt/wt | m | n a | n a | 2014 | 53 |  |
| 5 | ALS | *C9orf72*^exp^/wt | f | 53 | 29 | 2008 | 50 | Repeat size:  2050/5 |
| 6 | FTD | *C9orf72*^exp^/wt | m | 65 | >74* | 2011 | 43 | Repeat size:  2800/5 |
| 7 | ALS | *SOD1*^A4V^/wt | m | 62 | 16 | 2005 | 25 |  |
| 8 | ALS | *SOD1*^H46R^/wt | m | 54 | >143* | 2007 | 26 |  |
| 9 | Unaffected | *SOD1*^G127X^/wt | m | Asymp-tomatic | n a | 2007 | 24 | 5 aa neopeptide sequence following codon G127. 132 aa long |
| 10 | ALS | *SOD1*^E78_R79insSI^/wt | f | 42 | 20 | 2008 | 25 | Inserts 2 aa SI between exons 3 and 4. 155 aa long |
| 11 | ALS | *SOD1*^N86S^/wt | m | 77 | 24 | 2008 | 25 |  |
| 12 | ALS | *SOD1*^L117V^/wt | f | 36 | >116* | 2008 | 48 |  |
| 13 | ALS | *SOD1*^D90A/D90A^ | f | 52 | >324* | 2008 | 47 |  |
| 14 | ALS | *SOD1*^D125Tfs*24^/wt | m | 55 | >107* | 2008 | 26 | 23 aa neopeptide sequence following codon D124. 147 aa long |
| 15 | ALS | *SOD1*^G93A^/wt | m | 42 | 11** | 2012 | 28 |  |

^31 year old asymptomatic carrier of the *SOD1* G127X mutation.

*Living with the disease duration listed (census June 2015).

**11 months following onset of symptoms. Received a tracheostomy and placed on permanent invasive ventilation due to respiratory failure. Living 47 months after disease onset.

*** SOD1 activity in erythrocytes derived from patient blood samples.
